# Depression and Anxiety in Adolescents During the COVID-19 Pandemic in Relation to the Use of Digital Technologies: Longitudinal Cohort Study

**DOI:** 10.2196/45114

**Published:** 2024-02-07

**Authors:** Chen Shen, Rachel B Smith, Joel Heller, Alexander D V Spiers, Rhiannon Thompson, Helen Ward, Jonathan P Roiser, Dasha Nicholls, Mireille B Toledano

**Affiliations:** 1 MRC Centre for Environment and Health School of Public Health Imperial College London London United Kingdom; 2 National Institute for Health Research Health Protection Research Unit in Chemical and Radiation Threats and Hazards Imperial College London London United Kingdom; 3 Mohn Centre for Children’s Health and Wellbeing School of Public Health Imperial College London London United Kingdom; 4 National Institute for Health Research School for Public Health Research Imperial College London London United Kingdom; 5 Patient Experience Research Centre School of Public Health Imperial College London London United Kingdom; 6 Institute of Cognitive Neuroscience University College London London United Kingdom; 7 Department of Brain Sciences Division of Psychiatry Imperial College London London United Kingdom

**Keywords:** COVID-19, depression, anxiety, public health, adolescence, digital technology use, sleep, mobile phone

## Abstract

**Background:**

Adolescents are susceptible to mental illness and have experienced substantial disruption owing to the COVID-19 pandemic. The digital environment is increasingly important in the context of a pandemic when in-person social connection is restricted.

**Objective:**

This study aims to estimate whether depression and anxiety had worsened compared with the prepandemic period and examine potential associations with sociodemographic characteristics and behavioral factors, particularly digital behaviors.

**Methods:**

We analyzed cross-sectional and longitudinal data from a large, representative Greater London adolescent cohort study: the Study of Cognition, Adolescents and Mobile Phones (SCAMP). Participants completed surveys at T1 between November 2016 and July 2018 (N=4978; aged 13 to 15 years) and at T2 between July 2020 and June 2021 (N=1328; aged 16 to 18 years). Depression and anxiety were measured using the Patient Health Questionnaire and Generalized Anxiety Disorder scale, respectively. Information on the duration of total mobile phone use, social network site use, and video gaming was also collected using questionnaires. Multivariable logistic regression was used to assess the cross-sectional and longitudinal associations of sociodemographic characteristics, digital technology use, and sleep duration with clinically significant depression and anxiety.

**Results:**

The proportion of adolescents who had clinical depression and anxiety significantly increased at T2 (depression: 140/421, 33.3%; anxiety: 125/425, 29.4%) compared with the proportion of adolescents at T1 (depression: 57/421, 13.5%; anxiety: 58/425, 13.6%; *P* for 2-proportion *z* test <.001 for both depression and anxiety). Depression and anxiety levels were similar between the summer holiday, school opening, and school closures. Female participants had higher odds of new incident depression (odds ratio [OR] 2.5, 95% CI 1.5-4.18) and anxiety (OR 2.11, 95% CI 1.23-3.61) at T2. A high level of total mobile phone use at T1 was associated with developing depression at T2 (OR 1.89, 95% CI 1.02-3.49). Social network site use was associated with depression and anxiety cross-sectionally at T1 and T2 but did not appear to be associated with developing depression or anxiety longitudinally. Insufficient sleep at T1 was associated with developing depression at T2 (OR 2.26, 95% CI 1.31-3.91).

**Conclusions:**

The mental health of this large sample of adolescents from London deteriorated during the pandemic without noticeable variations relating to public health measures. The deterioration was exacerbated in girls, those with preexisting high total mobile phone use, and those with preexisting disrupted sleep. Our findings suggest the necessity for allocating resources to address these modifiable factors and target high-risk groups.

## Introduction

### Background

The global COVID-19 pandemic was an extraordinary public health crisis, involving unprecedented public health measures such as social distancing and school and business closures. On March 23, 2020, the UK government announced a national lockdown and urged the public to stay at home to reduce the spread of COVID-19. Lockdown restrictions were relaxed during the summer (May to August 2020), allowing for some easing of social measures, such as permitting 2 households to meet while adhering to social distancing guidelines. The lockdown was reintroduced in winter 2020 to mitigate the increasing transmission and protect the health care system from being overwhelmed. However, such measures may put people at risk of depression, anxiety, stress, and helplessness [1,2].

Adolescence is a life stage when individuals are susceptible to the onset of mental illness, of which depression and anxiety are most common. Therefore, adolescents may have been more susceptible to the mental health impacts of the public health crisis than adults. A United Kingdom–based survey found that 80% of the respondents believed that the pandemic had worsened their mental health, and 67% of the respondents believed that the pandemic would have a long-term negative effect on their mental health [3]. Adolescence is a critical period when peer relationships become more influential than family relationships on their life [4]. Social isolation from peers and loneliness are related to depressive symptoms, suicidal ideation, and anxiety [5]. In addition, school closures were associated with fear, restlessness, and sadness [6]. However, whether the impacts of the pandemic on mental health were acutely related to public health measures (eg, mental health status improves as restrictions ease) or enduring (eg, persistently poor mental health status during the pandemic) remains unclear. Understanding these impacts remains a policy priority to protect and improve young people’s mental health [7].

Use of mobile phones and other wireless devices is ubiquitous among children and adolescents. In the United Kingdom, 97% of the adolescents aged 12 to 15 years and 100% of the adolescents aged 16 to 17 years own a mobile phone, and most of them use a mobile phone, tablet, or laptop to go to the web [8]. Our recent systematic review found suggestive but limited evidence indicating associations between greater technology use and poorer mental health in children and adolescents [9]. More high-quality longitudinal studies with detailed information on digital technology use are needed, particularly in the context of the COVID-19 pandemic when the digital environment is increasingly important for learning, connection, and social support to offset the negative impacts of school closures and social isolation on mental health.

Previous cross-sectional studies found a high prevalence of depression and anxiety in young people during the pandemic [10-12]. Female gender, older age, and lower socioeconomic status (SES; eg, financial strain and living in rural areas) were associated with more mental health problems [13]. However, it is difficult to assess the impacts of the pandemic without information on the prepandemic symptom levels for comparison. In addition, other factors related to mental health including increased digital technology use (particularly the use of social network sites [SNSs]) and more sleep problems were reported during the pandemic [14,15]. Given that the associations between such behaviors and mental health are complex and often bidirectional, longitudinal analysis is necessary to unravel this interrelationship. A few longitudinal studies in adolescents have investigated depression and anxiety before and during the pandemic with mixed results [16-18]. One study reported an increase in depression and anxiety [16]. One study found an increase in depression, but anxiety remained stable both before and during the pandemic [17]. Another survey in school Year 9 students (the third year of secondary school; aged 13 years) found a decrease in anxiety during the UK lockdown [18]. However, older adolescents in critical examination years (General Certificate of Secondary Education [at the end of compulsory secondary education, aged approximately 16 years] and advanced level [for university admissions, aged approximately 18 years] examinations) may experience more stress owing to missing school time, as the potential impact on future university and employment opportunities is more immediate. However, none of these studies examined the longitudinal associations between digital technology use, sleep, and mental health.

### Objectives

This study aims to investigate the impact of the COVID-19 pandemic on adolescents’ mental health in a large representative (in terms of gender, ethnicity, and SES) longitudinal adolescent cohort across Greater London (the Study of Cognition, Adolescents and Mobile Phones [SCAMP]), for whom there was detailed information on mental and physical health and digital behaviors such as mobile phone use, SNS use, and video gaming, both before and during the pandemic. Thus, we were in a unique position to assess and tease out potential relationships between depression and anxiety, sociodemographic characteristics, digital technology use, sleep, and COVID-19 infection status and ascertain impacts in this important age group specifically related to the pandemic and public health measures. This will help to identify the groups at greatest risk who may benefit from targeted support.

## Methods

### Participants

The SCAMP is a prospective adolescent cohort study that was originally set up to investigate cognitive and behavioral outcomes affected by the use of mobile phones and other wireless technologies that emit radio-frequency electromagnetic fields. The details of this study have been reported previously [19]. Between November 2014 and July 2016, baseline data were collected from 6581 participants in Year 7 (aged 11 to 12 years) from 39 secondary schools (26 state and 13 independent) in and around Greater London, the United Kingdom. Participants in all SCAMP schools completed a computer-based assessment using the Psytools software (Delosis Ltd) under examination-like conditions in school. The assessment included a questionnaire on their digital technology behaviors (eg, smartphone use, SNS engagement, and video gaming); a battery of cognitive tests; and physical and mental health, lifestyle, and behavior scales. Data collected from 4978 participants at 31 schools between November 2016 and July 2018 (T1) when they were in Year 9 or 10 (aged 13 to 15 years) were included in this study. Participants who had depression (n=3292) and anxiety measures (n=3350) were defined as the T1 cross-sectional sample.

All SCAMP participants were invited to complete another assessment between July 2020 and June 2021 (T2) when they were aged 16 to 18 years, comprising cognitive tests and questionnaires on mental health, digital technology behaviors, home and outdoor environment, lifestyle, and access to public health information. Participants completed the assessment at home from July to September 2020. The assessment from September 2020 onward was conducted in school, supervised by the SCAMP team onsite or remotely via internet depending on school policies. Data collected from 1328 adolescents at T2 were included in this study. Participants who had depression and anxiety measures (n=968) were defined as the T2 cross-sectional sample.

**Figure 1:**
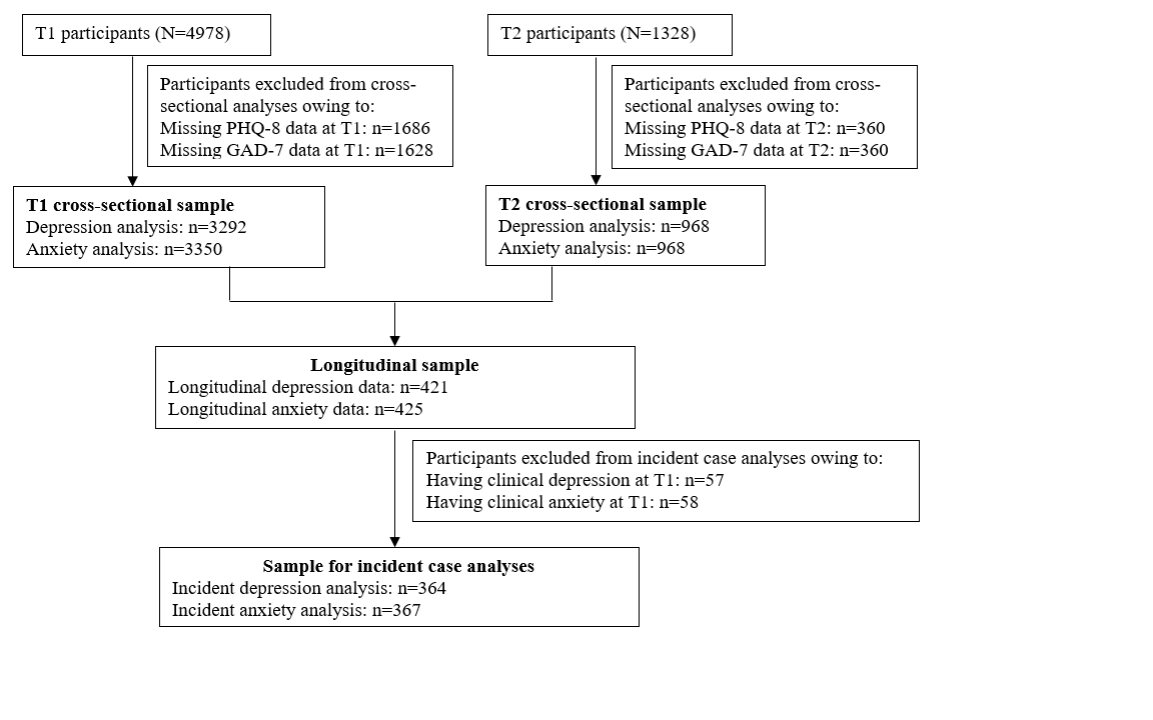
Structure of the Study of Cognition, Adolescents and Mobile Phones (SCAMP) cohort data relevant to this study. GAD-7: 7-item Generalized Anxiety Disorder; PHQ-8: 8-item Patient Health Questionnaire; T1: November 2016 to July 2018; T2: July 2020 to June 2021.

A subset of the participants had longitudinal depression (n=421) and anxiety (n=425) data, defined as the longitudinal sample. In the longitudinal sample, we excluded participants with clinically significant symptoms at T1 when assessing the longitudinal associations between the exposure variables and new incident depression and anxiety. The remaining participants were defined as the sample for incident case analysis (n=364 for incident depression analysis and n=367 for incident anxiety analysis). The sample for incident case analysis had 80% power to detect odds ratio (OR) of 1.82 and 1.84 when assessing the associations between digital technology use (eg, total mobile phone use) in tertile categories and incident depression and anxiety, respectively. Figure 1 shows the structure of the SCAMP cohort data relevant to this study.

### Measures (Self-Reported Questionnaire Data)

#### Sociodemographic Characteristics

Sociodemographic information including age, gender, ethnicity, and parental occupation was collected. The occupation of both parents was assessed at T1 only. We used the Office for National Statistics classification of occupation, categorizing it as managerial and professional, intermediate, and routine and manual [20]. The participants were assigned the higher parental occupation level. We also assessed the parental job situation since the COVID-19 lockdown at T2. We dichotomized the parental job situation as change during the pandemic and no change. School type (independent or state) was also included in the analyses. Missing data on ethnicity (262/4978, 5.26%), parental occupation (691/4978, 13.88%), and parental job situation (328/1328, 24.7%) were assigned a “missing” category for each variable rather than being excluded from the analyses.

#### Digital Technology Use (Total Mobile Phone Use, SNS Use, and Video Gaming)

At T1, participants reported the daily duration of phone calls and internet use (eg, web browsing, WhatsApp, Facebook, YouTube, and any other web-based apps) on mobile phones separately for weekdays and weekends. At T1, total mobile phone use was calculated as the combined daily duration of phone calls and internet use on mobile phones, with the average taken over both weekdays and weekends. At T2, participants reported an average daily duration of total mobile phone use for any purpose in the previous week.

At T1, participants reported the daily duration of SNS (eg, Facebook, Instagram, and Twitter) use on mobile phones and other devices separately for weekdays and weekends. At T1, SNS use was calculated as the combined daily duration of SNS use on mobile phones and other devices, with the average taken over both weekdays and weekends. At T2, participants reported the average time spent on specific SNS platforms (regardless of device) per day, including Facebook, Instagram, Twitter, TikTok, Snapchat, and YouTube in the last week. SNS use at T2 was calculated as the combined duration of SNS use on different platforms.

At T1, participants reported the daily duration of playing video games on any device at T1 separately for weekdays and weekends. Video gaming at T1 was the average weekday and weekend daily duration. At T2, participants reported an average daily duration of playing video games on any device in the last week.

Categorical responses were provided for questions on total mobile phone use, SNS use, and video gaming. To enable calculation and combination, we took the midpoints of category intervals, except in the highest category where we used the lowest value. For instance, the category “11-30 minutes per day” was converted to 20.5 minutes per day or 0.34 hours per day, whereas “more than 5 hours per day” was converted to 5 hours per day. Details for response category intervals for each question are shown in Table S1 in Multimedia Appendix 1. Participants were categorized into 3 groups based on the tertiles of daily duration of total mobile phone use, SNS use, and video gaming at each time point.

#### Sleep

At T1, participants provided information on when they usually got into bed, how long it took them to fall asleep, and what time they usually woke up separately for weekdays and weekends. The sleep duration was derived from these responses. The details have been reported elsewhere [21]. At T2, participants reported an average duration of sleep each night in the past 4 weeks. At T1 and T2, a sleep duration of <7 hours and >10 hours per day was defined as insufficient sleep and oversleep, respectively [22].

#### COVID-19 Infection Status

At T2, participants were asked if they had had a confirmed (by a positive test) or suspected COVID-19 infection (by a physician or themselves, but not tested).

#### Mental Health Outcomes—Depression and Anxiety

Depression was assessed using the 8-item Patient Health Questionnaire (PHQ-8). A summary score of ≥10 is considered indicative of clinically significant depression [23]. Cronbach α for the PHQ-8 score was .86 in the T1 cross-sectional sample and .88 in the T2 cross-sectional sample, indicating good internal reliability. To our knowledge, no previous study has examined the validity of the PHQ-8 in adolescents, but the PHQ-9 has demonstrated good validity in adolescents [24]. Anxiety was assessed using the 7-item Generalized Anxiety Disorder (GAD-7) scale. A summary score of ≥10 is considered indicative of clinically significant anxiety [25]. Cronbach α for the GAD-7 score was .89 in the T1 cross-sectional sample and .91 in the T2 cross-sectional sample, indicating good internal reliability. Evidence from current literature has indicated good validity and reliability of the GAD-7 in adolescents [26].

### Statistical Analyses

We tested the significance of the difference between the T1 and T2 time-varying factors in participants with data at both time points. We performed multivariable logistic regression in T1 and T2 cross-sectional samples to assess the associations of sociodemographic characteristics, digital technology use, and sleep with clinically significant depression and anxiety at ages 13 to 15 years and 16 to 18 years. In the T2 cross-sectional sample, we also assessed whether mental health differed by public health measures (eg, lockdown, school closures, and school reopening), using time as a proxy. We categorized it as summer holiday (July to August 2020), school opening (September to December 2020 and March to June 2021), and school closures (January to February 2021). To assess whether the associations between COVID-19 infection and mental health were attributed to residual confounding bias, we used depression and anxiety at T1 as the negative control dependent variables. We expected no significant association between COVID-19 infection status at T2 and depression or anxiety at T1.

Multivariable logistic regression was performed to assess longitudinal associations of sociodemographic characteristics, digital technology use, and sleep with new incident depression and anxiety at T2 in the sample for incident case analysis. Multiple logistic regression was used to yield ORs and 95% CIs. All variables were mutually adjusted in the models to examine the associations between sociodemographic factors and depression and anxiety. Depression and anxiety associated with total mobile phone use, SNS use, video gaming, sleep, and COVID-19 infection status were analyzed separately for each exposure variable (to avoid potential collider bias) by adjusting for all sociodemographic variables.

We conducted sensitivity analyses to examine cross-sectional associations between sociodemographic variables, digital technology use, sleep, COVID-19 infection status, and mental health outcomes at T1 and T2 in the longitudinal sample only. These analyses aimed to ascertain whether the associations observed in the longitudinal sample were substantially different from those observed in T1 and T2 cross-sectional samples. We performed an additional sensitivity analysis to investigate both cross-sectional and longitudinal associations between SNS use on mobile phones and depression and anxiety. This was done to determine if the patterns of association differed from those observed between SNS use on any device and mental health.

We did not use survey weights in our analyses, as the SCAMP is a school-based study that did not use probability sampling in participant recruitment. All analyses in this study were complete case analyses. Multiple imputation is not appropriate in our study, as it is probable that our data are missing not at random (ie, missingness is related to unobserved data). Participation in the T2 assessment might have been self-selected, given that data collection was mostly undertaken in a remote setting [27]. A *P* value of <.05 was considered statistically significant. All analyses were performed using R (version 4.0.3; R Foundation for Statistical Computing) and STATA (version 16.0; StataCorp).

### Ethical Considerations

The North-West Haydock Research Ethics Committee approved the SCAMP study protocol and its subsequent amendments (#14/NW/0347). School head teachers consented to participate in the SCAMP. Participants received written information about the study in advance and were given the option to withdraw from the research at any time. This study was conducted in accordance with the Declaration of Helsinki.

## Results

### Descriptive Statistics of the SCAMP Cohort

Table 1 shows that our sample was diverse in terms of sociodemographic characteristics. Table S2 in Multimedia Appendix 1 shows that the level of total mobile phone and SNS use significantly increased at T2 (*P* values for paired *t* test <.001). The prevalence of insufficient sleep also significantly increased at T2 (*P* value for 2-proportion *z* test <.001). The proportion of depression and anxiety significantly increased from 13.5% (57/421) at T1 to 33.3% (140/421) at T2 and from 13.6% (58/425) at T1 to 29.4% (125/425) at T2 (both *P* values for 2-proportion *z* test <.001), respectively.

**Table 1:** Sociodemographic characteristics, digital technology use, sleep, COVID-19 infection status, and depression and anxiety in the SCAMP cohort at T1^a^ and T2^b^.

Characteristics			T1 (n=4978)		T2 (n=1328)
Age (y), median (IQR)			14.02 (13.11-14.06)		17.06 (17.01-17.1)
**Gender, n (%)**					
	Male	2273 (45.66)		548 (41.27)	
	Female	2705 (54.34)		777 (58.51)	
	Missing	0 (0)		3 (0.23)	
**Ethnicity, n (%)**					
	Asian	1319 (26.5)		434 (32.68)	
	Black	728 (14.62)		128 (9.64)	
	White	2153 (43.25)		600 (45.18)	
	Other	516 (10.37)		166 (12.5)	
	Missing	262 (5.26)		0 (0)	
**Parental occupation^c^, n (%)**					
	Managerial and professional	2645 (53.13)		477 (35.92)	
	Intermediate	991 (19.91)		158 (11.9)	
	Routine or manual	651 (13.08)		82 (6.17)	
	Missing	691 (13.88)		611 (46.01)	
**School type, n (%)**					
	Independent	1287 (25.85)		459 (34.56)	
	State	3691 (74.15)		869 (65.44)	
**Parental job situation, n (%)**					
	No change since the lockdown	N/A^d^		786 (59.19)	
	Change since the lockdown	N/A		214 (16.11)	
	Missing	N/A		328 (24.7)	
**Time of data collection, n (%)**					
	Summer holiday	N/A		722 (54.37)	
	School opening	N/A		451 (33.96)	
	School closures	N/A		155 (11.67)	
Total mobile phone use (hours), mean (SD)			2.52 (2.13)		4.6 (2.43)
SNS^e^ use on any device (hours), mean (SD)			2.27 (2.69)		5.06 (3.82)
Video gaming on any device (hours), mean (SD)			1.12 (1.43)		1.15 (1.59)
**Sleep, n (%)**					
	Normal	2990 (60.06)		366 (27.56)	
	Insufficient	1469 (29.51)		505 (38.03)	
	Oversleep	265 (5.32)		27 (2.03)	
	Missing	254 (5.1)		430 (32.38)	
**COVID-19 infection status, n (%)**					
	No	N/A		807 (60.77)	
	Suspected infection	N/A		231 (17.39)	
	Confirmed diagnosis	N/A		37 (2.79)	
	Missing	N/A		253 (19.05)	
Prevalence of depression, % (n/N)			15.31 (504/3292)		30.89 (299/968)
Prevalence of anxiety, % (n/N)			13.22 (443/3350)		25.93 (251/968)

^a^T1: November 2016 to July 2018.

^b^T2: July 2020 to June 2021.

^c^Parental occupation was asked at T1 only; we assumed that parental occupation remained the same at T2.

^d^N/A: not applicable.

^e^SNS: social network site.

### Results From Cross-Sectional Analyses

Tables 2 and 3 show analyses in T1 and T2 cross-sectional samples. Table 2 shows that compared with male participants, female participants had higher odds of presenting depression (T1: OR 2.64, 95% CI 2.15-3.25; T2: OR 2.59, 95% CI 1.9-3.52) and anxiety (T1: OR 3.3, 95% CI 2.62-4.15; T2: OR 2.55, 95% CI 1.83-3.54) at both time points. At T1, older participants and those from state schools had higher odds of depression and anxiety than younger participants and those from independent schools. There were no associations between mental health measures and ethnicity or parental occupation at any time point. Depression and anxiety at T2 did not differ significantly between the summer holiday, school opening, and school closures. In sensitivity cross-sectional analyses restricted to the longitudinal sample, although there was some variation in OR values, the cross-sectional associations with gender persisted. However, the associations with age and school type at T1 were no longer statistically significant (Table S3 in Multimedia Appendix 1). It is worth noting that the smaller sample size in the longitudinal sample might have reduced the power to detect associations.

**Table 2:** The associations between sociodemographic factors^a^ and the presence of depression and anxiety in T1^b^ and T2^c^ cross-sectional samples.

Outcome and exposure			T1, OR^d^ (95% CI)	T2, OR (95% CI)
**Depression**				
	Age (per 1-y increase)		1.23 (1.02-1.48)	0.85 (0.66-1.11)
	**Gender**			
		Male	Reference	Reference
		Female	2.64 (2.15-3.25)	2.59 (1.9-3.52)
	**Ethnicity**			
		Asian	0.97 (0.76-1.24)	1.01 (0.71-1.44)
		Black	0.91 (0.66-1.27)	0.90 (0.51-1.57)
		White	Reference	Reference
		Other	1.20 (0.87-1.65)	0.82 (0.5-1.32)
	**Parental occupation**			
		Managerial and professional	Reference	Reference
		Intermediate	0.95 (0.74-1.22)	1.03 (0.64-1.67)
		Routine or manual	0.98 (0.73-1.32)	0.88 (0.44-1.77)
	**Parental job situation**			
		No change since the lockdown	N/A^e^	Reference
		Change since the lockdown	N/A	1.39 (0.97-1.98)
	**School type**			
		Independent	Reference	Reference
		State	1.49 (1.15-1.94)	1.41 (0.98-2.03)
	**Time of data collection**			
		Summer holiday	N/A	Reference
		School opening	N/A	0.74 (0.53-1.04)
		School closures	N/A	1.24 (0.79-1.94)
**Anxiety**				
	Age (per 1-y increase)		1.27 (1.04-1.55)	0.92 (0.7-1.2)
	**Gender**			
		Male	Reference	Reference
		Female	3.3 (2.62-4.15)	2.55 (1.83-3.54)
	**Ethnicity**			
		Asian	0.79 (0.61-1.04)	0.93 (0.64-1.35)
		Black	0.93 (0.66-1.31)	0.57 (0.30-1.07)
		White	Reference	Reference
		Other	1.34 (0.97-1.85)	0.83 (0.50-1.38)
	**Parental occupation**			
		Managerial and professional	Reference	Reference
		Intermediate	0.84 (0.64-1.1)	0.90 (0.54-1.51)
		Routine or manual	0.97 (0.71-1.33)	1.32 (0.66-2.62)
	**Parental job situation**			
		No change since the lockdown	N/A	Reference
		Change since the lockdown	N/A	1.06 (0.72-1.56)
	**School type**			
		Independent	Reference	Reference
		State	1.38 (1.05-1.81)	1.16 (0.79-1.68)
	**Time of data collection**			
		Summer holiday	N/A	Reference
		School opening	N/A	0.90 (0.63-1.27)
		School closures	N/A	0.74 (0.44-1.22)

^a^All sociodemographic variables were mutually adjusted at T1 and T2.

^b^T1: November 2016 to July 2018.

^c^T2: July 2020 to June 2021.

^d^OR: odds ratio.

^e^N/A: not applicable.

**Table 3:** The associations^a^ between digital technology use, sleep, COVID-19 infection status, and the presence of depression and anxiety in T1^b^ and T2^c^ cross-sectional samples.

Outcome and exposure				T1, OR^d^ (95% CI)		T2, OR (95% CI)
**Depression**						
	**Total mobile phone use^e^**					
		1st tertile	Reference		Reference	
		2nd tertile	1.12 (0.87-1.45)		1.26 (0.87-1.81)	
		3rd tertile	2.09 (1.64-2.65)		1.87 (1.3-2.69)	
	**SNS^f^ use on any device^g^**					
		1st tertile	Reference		Reference	
		2nd tertile	1.34 (1.04-1.74)		1.13 (0.78-1.64)	
		3rd tertile	1.97 (1.53-2.52)		1.63 (1.12-2.36)	
	**Video gaming on any device^h^**					
		1st tertile	Reference		Reference	
		2nd tertile	1.45 (1.14-1.85)		1.12 (0.79-1.6)	
		3rd tertile	1.82 (1.37-2.43)		1.44 (0.91-2.29)	
	**Sleep**					
		Normal	Reference		Reference	
		Insufficient	2.73 (2.22-3.34)		3.6 (2.52-5.14)	
		Oversleep	1.6 (1.02-2.49)		10.79 (4.12-28.29)	
	**COVID-19 infection status^i^**					
		No	Reference		Reference	
		Suspected infection	1.7 (0.9-3.21)		1.79 (1.28-2.5)	
		Confirmed diagnosis	0.61 (0.08-4.88)		0.59 (0.23-1.5)	
**Anxiety**						
	**Total mobile phone use^j^**					
		1st tertile	Reference		Reference	
		2nd tertile	1.36 (1.03-1.79)		1.16 (0.79-1.71)	
		3rd tertile	2.33 (1.8-3.03)		1.57 (1.06-2.3)	
	**SNS use on any device^k^**					
		1st tertile	Reference		Reference	
		2nd tertile	1.21 (0.92-1.57)		1.23 (0.83-1.83)	
		3rd tertile	1.57 (1.21-2.04)		1.91 (1.29-2.82)	
	**Video gaming on any device^l^**					
		1st tertile	Reference		Reference	
		2nd tertile	1.31 (1.02-1.69)		1.09 (0.75-1.57)	
		3rd tertile	1.44 (1.06-1.96)		1.09 (0.66-1.78)	
	**Sleep**					
		Normal	Reference		Reference	
		Insufficient	2.34 (1.89-2.9)		3.08 (2.12-4.46)	
		Oversleep	1.16 (0.7-1.92)		3.31 (1.32-8.3)	
	**COVID-19 infection status**					
		No	Reference		Reference	
		Suspected infection	1.67 (0.88-3.17)		1.93 (1.36-2.74)	
		Confirmed diagnosis	1.44 (0.3-6.96)		1.27 (0.54-2.96)	

^a^Mental health measures in relation to each exposure were analyzed separately by adjusting for the confounders as follows: T1 analysis: adjusted for age, gender, ethnicity, parental occupation, and school type; T2 analysis: adjusted for age, gender, ethnicity, parental occupation, parental job situation, school type, and time of data collection.

^b^T1: November 2016 to July 2018.

^c^T2: July 2020 to June 2021.

^d^OR: odds ratio.

^e^At T1, *P* for trend <.001; at T2, *P* for trend <.001.

^f^SNS: social network site.

^g^At T1, *P* for trend <.001; at T2, *P* for trend =.01.

^h^At T1, *P* for trend <.001; at T2, *P* for trend =.14.

^i^COVID-19 infection status reflects whether a participant has ever had a confirmed or suspected COVID-19 infection at any time before T2. Depression and anxiety at T1 were negative control outcomes when assessing the association between the COVID-19 infection status and mental health.

^j^At T1, *P* for trend <.001; at T2, *P* for trend =.03.

^k^At T1, *P* for trend <.001; at T2, *P* for trend =.001.

^l^At T1, *P* for trend *=*.01; at T2, *P* for trend =.69.

Table 3 shows that the total use of mobile phones and SNSs was associated with depression and anxiety at both time points with a dose-response relationship (all *P* values for trend <.05). Video gaming was associated with depression and anxiety at T1 with a dose-response relationship (both *P* values for trend <.05). Insufficient sleep was associated with depression (T1: OR 2.73, 95% CI 2.22-3.34; T2: OR 3.6, 95% CI 2.52-5.14) and anxiety (T1: OR 2.34, 95% CI 1.89-2.9; T2: OR 3.08, 95% CI 2.12-4.46) at both time points. Oversleep was associated with depression at both time points, with a stronger association at T2 (T1: OR 1.6, 95% CI 1.02-2.49; T2: OR 10.79, 95% CI 4.12-28.29). Suspected COVID-19 infection was associated with depression and anxiety at T2. This was not the case for confirmed COVID-19 infection; however, the low numbers of participants in this category (n<40) limit the sensitivity of this analysis. However, no significant associations between suspected or confirmed COVID-19 infection and depression or anxiety were observed at T1, as expected. In general, these cross-sectional associations were similar in the longitudinal sample, although some associations were no longer significant (Table S4 in Multimedia Appendix 1).

### Results From Longitudinal Analyses

After excluding participants with preexisting depression or anxiety at T1, female participants had higher odds of new incident depression (OR 2.5, 95% CI 1.5-4.18) and anxiety (OR 2.11, 95% CI 1.23-3.61) at T2 than male participants (Table 4). Compared with White ethnicity, ethnic minority was not associated with greater new incident depression or anxiety at T2. Black ethnicity was associated with a lower incidence of anxiety at T2 (OR 0.33, 95% CI 0.09-1.22), although the association was marginally significant. Longitudinal associations between age and school type and the incidence of depression and anxiety were not marked.

**Table 4:** The longitudinal associations between sociodemographic factors^a^ and the incidence of depression and anxiety at T2^b^ in the sample for incident case analysis.

Exposure		Incidence of depression at T2^c^, OR^d^ (95% CI)	Incidence of anxiety at T2^e^, OR (95% CI)
Age at T1^f^ (per 1-y increase)		0.79 (0.47-1.33)	0.81 (0.47-1.40)
Age at T2 (per 1-y increase)		0.82 (0.51-1.32)	0.70 (0.42-1.15)
**Gender**			
	Male	Reference	Reference
	Female	2.5 (1.5-4.18)	2.11 (1.23-3.61)
**Ethnicity**			
	Asian	0.76 (0.43-1.36)	0.89 (0.49-1.61)
	Black	0.57 (0.2-1.64)	0.33 (0.09-1.22)
	White	Reference	Reference
	Other	0.51 (0.2-1.27)	0.62 (0.23-1.64)
**Parental occupation**			
	Managerial and professional	Reference	Reference
	Intermediate	0.99 (0.52-1.89)	0.83 (0.41-1.7)
	Routine or manual	0.97 (0.41-2.29)	1.51 (0.64-3.59)
**School type**			
	Independent	Reference	Reference
	State	1.43 (0.78-2.65)	1.17 (0.61-2.22)

^a^All sociodemographic variables were mutually adjusted.

^b^T2: July 2020 to June 2021.

^c^Participants with clinically significant depression at T1 were excluded.

^d^OR: odds ratio.

^e^Participants with clinically significant anxiety at T1 were excluded.

^f^T1: November 2016 to July 2018.

Compared with the participants who used mobile phones in the lowest tertile, those in the highest tertile had higher odds of new incident depression at T2 (OR 1.89, 95% CI 1.02-3.49; Table 5). SNS use and video gaming at T1 were not associated with the development of depression or anxiety at T2. Moderate SNS users (ie, those in the 2nd tertile) had slightly lower odds of new incident depression at T2, although this association was not significant. The associations between SNS use on mobile phones and mental health outcomes were generally similar to those found between SNS use on any device and mental health outcomes (Table S5 in Multimedia Appendix 1). Insufficient sleep at T1 was also associated with new incident depression at T2 (OR 2.26, 95% CI 1.31-3.91).

**Table 5:** The longitudinal associations^a^ between digital technology use and sleep at T1^b^ and the incidence of depression and anxiety at T2^c^ in the sample for incident case analysis.

Exposure at T1		Incidence of depression at T2^d^, OR^e^ (95% CI)	Incidence of anxiety at T2^f^, OR (95% CI)
**Total mobile phone use^g^**			
	1st tertile	Reference	Reference
	2nd tertile	1.12 (0.64-1.97)	1.11 (0.62-2)
	3rd tertile	1.89 (1.02-3.49)	1.48 (0.77-2.84)
**SNS^h^ use on any device^i^**			
	1st tertile	Reference	Reference
	2nd tertile	0.72 (0.42-1.25)	0.80 (0.45-1.43)
	3rd tertile	0.92 (0.48-1.75)	0.90 (0.47-1.75)
**Video gaming on any device^j^**			
	1st tertile	Reference	Reference
	2nd tertile	1.42 (0.79-2.57)	1.55 (0.82-2.91)
	3rd tertile	1.32 (0.63-2.76)	1.8 (0.83-3.92)
**Sleep**			
	Normal	Reference	Reference
	Insufficient	2.26 (1.31-3.91)	1.14 (0.64-2.05)
	Oversleep	1.41 (0.45-4.45)	0.66 (0.17-2.49)

^a^Adjusted for age at T1 and T2, gender, ethnicity, parental occupation, and school type at T1.

^b^T1: November 2016 to July 2018.

^c^T2: July 2020 to June 2021.

^d^Participants with clinically significant depression at T1 were excluded.

^e^OR: odds ratio.

^f^Participants with clinically significant anxiety at T1 were excluded.

^g^For depression, *P* for trend =.05; for anxiety, *P* for trend =.26.

^h^SNS: social network site.

^i^For depression, *P* for trend =.62; for anxiety, *P* for trend =.68.

^j^For depression, *P* for trend =.37; for anxiety, *P* for trend =.11.

## Discussion

### Principal Findings

To our knowledge, this is the first study to investigate detailed digital technology use in relation to longitudinal changes in mental health from the prepandemic period in a large representative adolescent sample with high sociodemographic diversity. We are also the first to consider mental health associated with public health measures (eg, lockdown, school closures, and school reopening) during the pandemic. We identified clear increases in depression and anxiety symptoms reaching clinical thresholds in adolescents over the course of the COVID-19 pandemic compared with the prepandemic assessments in the same cohort. Female individuals were more likely to develop depression and anxiety during the pandemic than male individuals. Black adolescents were less likely to develop anxiety during the pandemic than White adolescents. Depression and anxiety levels did not differ significantly according to socioeconomic factors or public health measures. High mobile phone use and insufficient sleep in the prepandemic period were associated with new incident depression during the pandemic. SNS use and video gaming were associated with depression and anxiety cross-sectionally only. Suspected COVID-19 infection was associated with increased depression and anxiety.

### Comparison With Prior Work

Compared with previous longitudinal studies examining the impacts of the pandemic on adolescent mental health [16-18,28], our sample had greater ethnic and SES diversity, enabling us to examine the associations between ethnicity and SES and mental health. In addition, we investigated mental health related to various aspects of digital technology use and sleep both cross-sectionally and longitudinally to indicate potential risk factors. There were no marked variations in depression and anxiety based on public health measures, suggesting that mental health impacts are not contingent on immediate context but rather arise from broader and potentially more lasting societal and individual influences.

Our findings indicate a marked increase in the prevalence of depression and anxiety during the pandemic. This could be attributed to factors such as enforced stay-at-home measures and school closures, which hindered peer interactions for school-aged adolescents. Such interactions are crucial for maintaining good mental health [29]. However, our results should be interpreted with caution, as the most recent data collection wave before the pandemic commenced when participants were aged 13 to 15 years (2016 to 2018) and still at a developmental stage when mental health problems begin to manifest. Our findings were comparable with another survey in a nationally representative sample, which also found that the rates of a probable mental health disorder increased from 1 in 10 in 2017 to 1 in 6 in 2021 among adolescents aged 17 to 19 years [28]. Moreover, we found that older age was not associated with new incident depression or anxiety during the pandemic (when participants were aged 16 to 18 years), suggesting the impacts of pandemic may have a more substantial influence on mental health decline than age itself.

In line with the findings of other studies that indicate gender differences in the prevalence of depression and anxiety during adolescence [30,31], we found that female gender was associated with depression and anxiety at both time points. Cognitive vulnerabilities, biological mechanisms (eg, sex hormones), and psychosocial factors may explain these differences [32-34]. We found that compared with male participants, female participants had a higher incidence of depression and anxiety during the pandemic, which echoes a recent longitudinal study [16]. This is perhaps because girls are more prone to rely on social support from their peer networks to cope with stressful life events [35]. Physical isolation and restricted interaction with peers, teachers, and the community posit a negative effect on coping strategies commonly used by girls [36]. However, another large UK population–based adolescent cohort study before the pandemic showed earlier and steeper increases in depressive symptoms in girls than in boys over the secondary school years [37], indicating that both usual gender-specific mental health trajectories and the impacts of the pandemic may underlie our findings.

Our findings on mental health differences by ethnicity are inconsistent with the hypothesized mental health vulnerability of ethnic minority groups in the general population [38]. One possible reason is that ethnic minority groups are clustered in some schools; thus, the potential compounding effects of social marginalization are not seen. In addition, Black ethnicity has been associated with higher psychological resilience during the pandemic [39], possibly explaining the lower incidence of anxiety in Black participants. It is also possible that our measures of anxiety and depression have measurement variance with respect to ethnicity or gender, reflecting limitations in how the items capture symptoms across groups (eg, owing to language) rather than differences in symptoms themselves [40,41].

We found new incident depression associated with total mobile phone use but not with SNS use at T1. The same levels of total mobile phone use and SNS use were associated with depression cross-sectionally at T1 in the T1 cross-sectional sample and longitudinal sample, corroborating our findings that the lack of longitudinal association between SNS use and depression is unlikely to be attributed to reduced power. In addition, we found that moderate SNS use was associated with slightly lower odds of new incident depression, although the association was not significant. Social networks established before the pandemic and moderate use of technology to maintain social connection remotely may be an important means to mitigate social isolation during lockdown. Adolescence is a sensitive period when social connection is particularly vital for brain development and mental health [42]. We did not find an association between video gaming and new incident depression or anxiety at T2. Although video gaming is associated with more depressive symptoms and behavioral difficulties in adolescents, web-based video gaming has a social component, that is, players are connected to each other, which might also mitigate social isolation during lockdown [43,44]. However, high mobile phone use may also carry the risk of displacement of sleep [45], enhancing social comparison [46], driving perfectionism [47], and increasing potential exposure to cyberbullying including social exclusion [48]. In addition, the short-term increase in screen time during the pandemic or the maladaptive technology use driven by poor mental health may explain the cross-sectional associations because of the possible bidirectional relationship between digital technology use and mental health. Our study found that insufficient sleep was associated with depression both cross-sectionally and longitudinally, indicating that insufficient sleep may be both a risk factor and a symptom of depression. It is also possible that individuals with preexisting sleep problems are more susceptible to the impact of adverse life experiences on mental health. Although COVID-19 infection suspected by the participants was associated with depression and anxiety, this requires further investigation with COVID-19 test results to confirm the infection status and its effect on adolescent mental health, as our sample of confirmed positive tests was low.

### Strengths and Limitations

Our study has strengths and limitations. Strengths include that this was a large-scale adolescent longitudinal study using an ethnically and socioeconomically diverse cohort and detailed data on mental health measures, lifestyle, and sociodemographic characteristics. The cohort is representative of school-aged adolescents across Greater London. The longitudinal study design with the same mental health measures before and during the pandemic enabled the investigation of changes in depression and anxiety symptoms and new incident depression and anxiety and clearly demonstrated the worsening of mental health over this public health crisis. The limitations of this study include the loss of follow-up from T1 to T2 owing to logistical difficulties in data collection during the pandemic, which reduced our power to undertake more detailed analyses in subgroups. However, the association patterns in the longitudinal sample were generally similar to those observed in T1 and T2 cross-sectional samples, indicating that selection bias owing to loss of follow-up is unlikely to be a major concern. The use of self-reported questionnaires to collect information on digital technology use, sleep, and COVID-19 infection status in our study was subject to recall bias. Questionnaires on digital technology use were different between T1 and T2, making direct comparisons for each question challenging. We only measured the duration of SNS use in our study. However, the mental health impact of SNSs could stem from the content adolescents interact with, or the type of interactions developed on the web, rather than the duration of SNS use itself. Given that the COVID-19 pandemic was a universal exposure, it is difficult to attribute the increase in mental health symptoms solely to the pandemic.

### Public Health Implications

This study has several public health implications. Worsened mental health and a high proportion of clinical depression and anxiety in adolescents during the pandemic require more mental health support beyond clinical services (eg, school-level and digitally delivered approaches) for young people, especially for girls. For instance, there may be some good options for girls to gain social support by connecting with their social networks via moderate use of SNSs to cope with stressful situations. However, schools, parents, and adolescents should be aware of the potential adverse effects of digital technology overuse on mental health and better manage digital activities. In addition, tackling sleep problems might mitigate the impacts of the pandemic on mental health in terms of prevention and early treatment. No significant impacts of contemporaneous public health measures on mental health were observed.

### Future Work

Future work should follow the SCAMP participants for more time to investigate the long-term effects on mental health. Objective data on screen time and sleep are needed in the future to minimize social desirability bias and measurement error from self-reported information. Future research should incorporate qualitative methodology to explore the reasons behind the varying mental health experiences among young people—some experience deterioration whereas others display resilience. This approach can provide insights to support those currently in need and mitigate the impact of future crises.

### Conclusions

Our findings show longitudinal increases in the prevalence of clinically significant depression and anxiety during the COVID-19 pandemic compared with those measured in the prepandemic period. However, variations in depression and anxiety relating to public health measures were not evident, suggesting that the impacts on adolescent mental health are enduring regardless of the immediate context. Girls, those with preexisting high total mobile phone use, and those with preexisting insufficient sleep had higher risks of developing new depression during the pandemic. Girls also had higher risks of developing new anxiety. Ethnic minority groups were not associated with higher levels of depression or anxiety. Our findings indicate that resources to target these modifiable factors and high-risk groups are needed. It is possible that these adverse effects on mental health will have a lasting impact. Therefore, we recommend continued surveillance of adolescents’ mental health to aid adequate provision of mental health services to this susceptible group of the population.

## Appendix

Multimedia Appendix 1 Details of digital technology use variables (Table S1), comparison of T1 (November 2016 to July 2018) and T2 (July 2020 to June 2021) time-varying factors (Table S2), and results from sensitivity analyses (Tables S3 to S5).

## Abbreviations

GAD-7: 7-item Generalized Anxiety Disorder
OR: odds ratio
PHQ-8: 8-item Patient Health Questionnaire
SCAMP: Study of Cognition, Adolescents and Mobile Phones
SES: socioeconomic status
SNS: social network site
