# Supplementary material for: Depression and Anxiety in Adolescents During the COVID-19 Pandemic in Relation to the Use of Digital Technologies: Longitudinal Cohort Study
Source: J Med Internet Res. 2024 Feb 7;26:e45114. doi: 10.2196/45114 (PMC10882466; doi:10.2196/45114)
Supplement: Multimedia Appendix 1 [file jmir_v26i1e45114_app1.docx]

Table S1 Response category intervals for self-reported digital technology use at T1^a^ and T2^b^

| **Digital technology use (T1)^c^** | **Questions** | **Response categories** |
| --- | --- | --- |
| Total mobile phone use | How much time per day do you spend  talking on your mobile phone? (on weekdays; on a weekend day) | None; 1 - 5 minutes; 6 - 15 minutes; 16 - 30 minutes; 31 - 59 minutes; 1 - 2 hours; 3 hours or more |
|  | How much time per day do you spend using the internet for any purpose on your mobile phone? (on weekdays; on a weekend day) | None; 1- 10 minutes; 11 - 30 minutes; 31 - 59 minutes; 1 - 2 hours; 3-4 hours; 5-6 hours; 7 hours or more |
| SNS^d^ use | How much time per day do you spend on social network sites on a mobile phone (on weekdays; on a weekend day)? | None; 1- 10 minutes; 11 - 30 minutes; 31 - 59 minutes; 1 - 2 hours; 3 - 4 hours; 5 hours or more |
|  | How much time per day do you spend on social network sites on other devices (on weekdays; on a weekend day) | None; 1- 10 minutes; 11 - 30 minutes; 31 - 59 minutes; 1 - 2 hours; 3 - 4 hours; 5 - 6 hours; 7 hours or more |
| Video gaming | How much time per day do you spend playing video games on any device? (on weekdays; on a weekend day) | None; 1- 10 minutes; 11 - 30 minutes; 31 - 59 minutes; 1 - 2 hours; 3 - 4 hours; 5 hours or more |
|  |  |  |
| **Digital technology use (T2)** | **Questions** | **Response categories** |
| Total mobile phone use | Thinking about the last week, how much time per day do you usually spend using  your smartphone? | 0 - 1 hour; 1 - 2 hours; 2 - 3 hours; 3 - 4 hours; 4 - 5 hours; 5 - 6 hours; 6 - 7 hours; 7 - 8 hours; 8 - 9 hours; 9 - 10 hours; More than 10 hours |
| SNS use | Thinking about the last week, how much time per day, do you usually spend on Facebook? | No time; 1 - 30 minutes; 31 - 59 minutes; 1 - 2 hours; 2 - 3 hours; 3 - 4 hours; 4 - 5 hours; 5 hours or more |
|  | Thinking about the last week, how much time per day, do you usually spend on Instagram? | No time; 1 - 30 minutes; 31 - 59 minutes; 1 - 2 hours; 2 - 3 hours; 3 - 4 hours; 4 - 5 hours; 5 hours or more |
|  | Thinking about the last week, how much time per day, do you usually spend on TikTok? | No time; 1 - 30 minutes; 31 - 59 minutes; 1 - 2 hours; 2 - 3 hours; 3 - 4 hours; 4 - 5 hours; 5 hours or more |
|  | Thinking about the last week, how much time per day, do you usually spend on Twitter? | No time; 1 - 30 minutes; 31 - 59 minutes; 1 - 2 hours; 2 - 3 hours; 3 - 4 hours; 4 - 5 hours; 5 hours or more |
|  | Thinking about the last week, how much time per day, do you usually spend on Snapchat? | No time; 1 - 30 minutes; 31 - 59 minutes; 1 - 2 hours; 2 - 3 hours; 3 - 4 hours; 4 - 5 hours; 5 hours or more |
|  | Thinking about the last week, how much time per day, do you usually spend on YouTube? | No time; 1 - 30 minutes; 31 - 59 minutes; 1 - 2 hours; 2 - 3 hours; 3 - 4 hours; 4 - 5 hours; 5 hours or more |
| Video gaming | Thinking about the last week, how much time per day do you spend playing video games on any device? | No time; 1 - 30 minutes; 31 - 59 minutes; 1 - 2 hours; 2 - 3 hours; 3 - 4 hours; 4 - 5 hours; 5 - 6 hours; 6 hours or more |

^a^T1: November 2016 to July 2018.

^b^T2: July 2020 to June 2021.

^c^At T1, each question was asked separately for weekdays and weekends with the same response categories.

^d^SNS: social network site.

Table S2 Comparison of T1^a^ and T2^b^ time-varying factors in participants with data at both timepoints

|  | T1 | T2 | *P* value |
| --- | --- | --- | --- |
| Total mobile phone use (hrs), mean (SD) | 2.42 (2.03) | 4.55 (2.4) | <.001^c^ |
| SNS^d^ use on any device (hrs), mean (SD) | 1.74 (2.22) | 4.93 (3.76) | <.001^c^ |
| Video gaming on any device (hrs), mean (SD) | 0.94 (1.29) | 1.09 (1.55) | <.01^c^ |
| **Sleep, n (%)** |  |  | <.001^e^ |
| Normal | 326 (67.5) | 193 (40) |  |
| Insufficient | 138 (28.6) | 275 (56.9) |  |
| Oversleep | 19 (3.9) | 15 (3.1) |  |
| Prevalence of depression, % (n/N) | 13.5 (57/421) | 33.3 (140/421) | <.001^e^ |
| Prevalence of anxiety, % (n/N) | 13.6 (58/425) | 29.4 (125/425) | <.001^e^ |

^a^T1: November 2016 to July 2018.

^b^T2: July 2020 to June 2021.

^c^Paired *t* test.

^d^SNS: social network site.

^e^2-proportion *z* test.

Table S3 The cross-sectional associations between socio-demographic factors^a^ and the presence of depression and anxiety at T1^b^ and T2^c^ in the longitudinal sample

| Outcome | Exposure | T1 | T2 |
| --- | --- | --- | --- |
|  |  | OR^d^ (95% CI) | OR (95% CI) |
| Depression | Age (per 1-y increase) | 1.03 (0.56-1.91) | 0.88 (0.57-1.36) |
|  | **Gender** |  |  |
|  | Male | Reference | Reference |
|  | Female | 2.64 (1.37-5.1) | 2.81 (1.76-4.49) |
|  | **Ethnicity** |  |  |
|  | Asian | 1.46 (0.72-2.95) | 0.94 (0.55-1.6) |
|  | White | Reference | Reference |
|  | Black | 1.15 (0.34-3.89) | 0.79 (0.31-1.99) |
|  | Other | 1.74 (0.69-4.39) | 0.43 (0.19-0.97) |
|  | **Parental occupation** |  |  |
|  | Managerial and professional | Reference | Reference |
|  | Intermediate | 1.05 (0.5-2.22) | 0.95 (0.53-1.71) |
|  | Routine or manual | 0.62 (0.21-1.85) | 0.72 (0.32-1.62) |
|  | **Parental job situation** |  |  |
|  | No change since lockdown | N/A^e^ | Reference |
|  | Change since lockdown | N/A | 1.68 (1-2.82) |
|  | **School type** |  |  |
|  | Independent | Reference | Reference |
|  | State | 1.36 (0.64-2.87) | 1.34 (0.77-2.36) |
|  | **Time of data collection** |  |  |
|  | Summer holiday | N/A | Reference |
|  | School opening | N/A | 0.65 (0.4-1.06) |
|  | School closures | N/A | 1 (0.5-2.02) |
| Anxiety | Age (per 1-y increase) | 1.05 (0.56-1.95) | 0.7 (0.45-1.08) |
|  | **Gender** |  |  |
|  | Male | Reference | Reference |
|  | Female | 3.12 (1.57-6.22) | 2.51 (1.55-4.06) |
|  | **Ethnicity** |  |  |
|  | Asian | 0.81 (0.39-1.68) | 0.83 (0.48-1.45) |
|  | Black | 0.89 (0.27-2.98) | 0.48 (0.18-1.33) |
|  | White | Reference | Reference |
|  | Other | 1.98 (0.84-4.69) | 0.68 (0.31-1.46) |
|  | **Parental occupation** |  |  |
|  | Managerial and professional | Reference | Reference |
|  | Intermediate | 1.28 (0.59-2.79) | 0.92 (0.5-1.69) |
|  | Routine or manual | 1.13 (0.4-3.23) | 1.37 (0.62-2.99) |
|  | **Parental job situation** |  |  |
|  | No change since lockdown | N/A | Reference |
|  | Change since lockdown | N/A | 1.12 (0.65-1.93) |
|  | **School type** |  |  |
|  | Independent | Reference | Reference |
|  | State | 1.04 (0.5-2.17) | 1.03 (0.58-1.82) |
|  | **Time of data collection** |  |  |
|  | Summer holiday | N/A | Reference |
|  | School opening | N/A | 0.92 (0.57-1.5) |
|  | School closures | N/A | 0.83 (0.39-1.76) |

^a^All sociodemographic variables were mutually adjusted at T1 and T2.

^b^T1: November 2016 to July 2018.

^c^T2: July 2020 to June 2021.

^d^OR: odds ratio.

^e^N/A: Not applicable.

Table S4 The cross-sectional associations^a^ between digital technology use, sleep, COVID-19 infection status, and the presence of depression and anxiety at T1^b^ and T2^c^ in the longitudinal sample

| Outcome | Exposure | T1 | T2 |
| --- | --- | --- | --- |
|  |  | OR^d^ (95% CI) | OR (95% CI) |
| Depression | **Total mobile phone use^e^** |  |  |
|  | 1^st^ tertile | Reference | Reference |
|  | 2^nd^ tertile | 1.75 (0.81-3.77) | 1.53 (0.87-2.69) |
|  | 3^rd^ tertile | 3.37 (1.56-7.26) | 2.6 (1.49-4.55) |
|  | **SNS^f^ use on any device^g^** |  |  |
|  | 1^st^ tertile | Reference | Reference |
|  | 2^nd^ tertile | 1.14 (0.56-2.31) | 1.52 (0.86-2.7) |
|  | 3^rd^ tertile | 2.27 (1.09-4.71) | 2.96 (1.66-5.3) |
|  | **Video gaming on any device^h^** |  |  |
|  | 1^st^ tertile | Reference | Reference |
|  | 2^nd^ tertile | 0.6 (0.28-1.26) | 1.52 (0.89-2.61) |
|  | 3^rd^ tertile | 0.95 (0.39-2.31) | 1.53 (0.75-3.11) |
|  | **Sleep** |  |  |
|  | Normal | Reference | Reference |
|  | Insufficient | 4.62 (2.51-8.49) | 3.21 (1.9-5.43) |
|  | Oversleep | 1.75 (0.37-8.34) | 20.18 (3.86-105.56) |
|  | **COVID-19 infection status^i^** |  |  |
|  | No | Reference | Reference |
|  | Suspected infection | 1.74 (0.9-3.36) | 2 (1.2-3.33) |
|  | Confirmed diagnosis | 0.66 (0.08-5.37) | 0.39 (0.08-1.91) |
| Anxiety | **Total mobile phone use^j^** |  |  |
|  | 1^st^ tertile | Reference | Reference |
|  | 2^nd^ tertile | 1.37 (0.64-2.97) | 1.33 (0.75-2.34) |
|  | 3^rd^ tertile | 3.27 (1.54-6.92) | 2.17 (1.24-3.8) |
|  | **SNS use on any device^k^** |  |  |
|  | 1^st^ tertile | Reference | Reference |
|  | 2^nd^ tertile | 0.95 (0.5-1.83) | 1.67 (0.94-2.97) |
|  | 3^rd^ tertile | 0.76 (0.35-1.66) | 2.53 (1.4-4.57) |
|  | **Video gaming on any device^l^** |  |  |
|  | 1^st^ tertile | Reference | Reference |
|  | 2^nd^ tertile | 0.7 (0.34-1.45) | 1.74 (1.02-2.99) |
|  | 3^rd^ tertile | 0.98 (0.4-2.41) | 1.26 (0.6-2.63) |
|  | **Sleep** |  |  |
|  | Normal | Reference | Reference |
|  | Insufficient | 4.32 (2.35-7.92) | 2.73 (1.62-4.61) |
|  | Oversleep | 1.69 (0.35-8.14) | 6.79 (1.87-24.71) |
|  | **COVID-19 infection status** |  |  |
|  | No | Reference | Reference |
|  | Suspected infection | 1.64 (0.85-3.16) | 2.12 (1.27-3.54) |
|  | Confirmed diagnosis | 1.42 (0.29-6.96) | 1.23 (0.34-4.43) |

^a^Mental health measures in relation to each exposure were analysed separately by adjusting for confounders as follows: T1 analysis: Adjusted for age, gender, ethnicity, parental occupation, and school type and T2 analysis: Adjusted for age, gender, ethnicity, parental occupation, parental job situation, school type, and time of data collection.

^b^T1: November 2016 to July 2018.

^c^T2: July 2020 to June 2021.

^d^OR: odds ratio.

^e^At T1, *P* for trend *=*.002 and at T2, *P* for trend <.001.

^f^SNS: social network site.

^g^At T1, *P* for trend *=*.033 and at T2, *P* for trend <.001.

^h^At T1, *P* for trend *=*.65 and at T2, *P* for trend =.18.

^i^COVID-19 infection status reflects whether a participant has ever had a confirmed or suspected COVID-19 infection at any time before T2. Depression and anxiety at T1 were negative control outcomes when assessing the association between the COVID-19 infection status and mental health.

^j^At T1, *P* for trend *=*.002 and at T2, *P* for trend =.007.

^k^At T1, *P* for trend *=*.52 and at T2, *P* for trend =.002.

^l^At T1, *P* for trend *=*.74 and at T2, *P* for trend =.31.

Table S5 Cross-sectional and longitudinal associations between SNS^a^ use on mobile phones at T1^b^ and depression and anxiety

| Outcome | SNS use on mobile phones at T1^c^ | Cross-sectional^d^ | Longitudinal^e^ |
| --- | --- | --- | --- |
|  |  | OR (95% CI) | OR (95% CI) |
| Depression^f^ | 1^st^ tertile | Reference | Reference |
|  | 2^nd^ tertile | 1.33 (1.03-1.72) | 0.84 (0.47-1.49) |
|  | 3^rd^ tertile | 1.82 (1.44-2.3) | 0.96 (0.52-1.76) |
| Anxiety^g^ | 1^st^ tertile | Reference | Reference |
|  | 2^nd^ tertile | 1.18 (0.9-1.54) | 1.25 (0.7-2.23) |
|  | 3^rd^ tertile | 1.43 (1.12-1.83) | 0.6 (0.31-1.19) |

^a^SNS: social network site.

^b^T1: November 2016 to July 2018.

^c^For depression, *P* for trend <.001 (cross-sectional analysis) and =.82 (longitudinal analysis). For anxiety, *P* for trend =.004 (cross-sectional analysis) and =.23 (longitudinal analysis).

^d^Adjusted for age, gender, ethnicity, parental occupation, and school type at T1.

^e^Adjusted for age at T1 and T2 (July 2020 to June 2021), gender, ethnicity, parental occupation, and school type at T1.

^f^Participants with clinically significant depression at T1 were excluded in the longitudinal analysis.

^g^Participants with clinically significant anxiety at T1 were excluded in the longitudinal analysis.
